# Lung microbiome in children with hematological malignancies and lower respiratory tract infections

**DOI:** 10.3389/fonc.2022.932709

**Published:** 2022-09-21

**Authors:** Yun Zhang, Haonan Ning, Wenyu Zheng, Jing Liu, Fuhai Li, Junfei Chen

**Affiliations:** ^1^ Department of Pediatrics, Qilu Hospital of Shandong University, Jinan, China; ^2^ Department of Biostatistics, School of Public Health, Cheeloo College of Medicine, Shandong University, Jinan, China; ^3^ Department of Pediatric Surgery, Qilu Hospital of Shandong University, Jinan, China

**Keywords:** lung microbiome, bronchoalveolar lavage fluid, children, hematological malignancies, lower respiratory tract infection, drug resistance, microbial tolerance, unfavorable environment

## Abstract

**Background:**

Respiratory infectious complications remain a major cause of morbidity and mortality in children with hematological malignancies. Knowledge regarding the lung microbiome in aforementioned children is limited.

**Methods:**

A prospective cohort was conducted, enrolling 16 children with hematological malignancies complicated with moderate-to-severe lower respiratory tract infections (LRTIs) versus 21 LRTI children with age, gender, weight, and infection severity matched, with no underlying malignancies, to evaluate the lung microbiome from bronchoalveolar lavage fluid samples in different groups.

**Results:**

The lung microbiome from children with hematological malignancies and LRTIs showed obviously decreased α and β diversity; increased microbial function in infectious disease:bacteria/parasite; drug resistance:antimicrobial and human pathogenesis than the control group; a significantly reduced proportion of *Firmicutes*, *Bacteroidota*, *Actinobacteriota*; increased *Proteobacteria* at the phylum level; and distinctly elevated *Parabacteroides*, *Klebsiella*, *Grimontia*, *Escherichia_Shigella*, *unclassified_Enterobacteriaceae* at the genus level than the control group. Furthermore, it was revealed that α diversity (Shannon), β diversity (Bray–Curtis dissimilarity), *Proteobacteria* at the phylum level, and *unclassified_Enterobacteriaceae* and *Escherichia_Shigella* at the genus level were significantly negatively associated with hospitalization course whereas *Firmicutes* at the phylum level was established positively correlated with the hospitalization course.

**Conclusions:**

Children with hematological malignancies and LRTIs showed obviously decreased α and β diversity, significantly increased function in infectious disease pathogenesis, antimicrobial drug resistance, and unfavorable environment tolerance. Moreover, α diversity (Shannon), β diversity (Bray–Curtis dissimilarity), and *Proteobacteria* may be used as negative correlated predictors for hospitalization course in these children whereas *Firmicutes* may be utilized as a positive correlated predictor.

## 1. Introduction

Over the past decades, major advances have been made in therapy for childhood hematological malignancies (1, 2), especially acute lymphoblastic leukemia, resulting in obviously higher 10-year survival rates than before (2–5). However, respiratory infectious complications remain a major cause of morbidity and mortality in the aforementioned children (3, 6–8). In particular, children undergoing chemotherapy with hematological malignancies are at a high risk of more severe infections with prolonged clinical treatment course and profound immunity condition.

Empirical antibiotherapy with wide-spectrum bactericidal drugs within early phase in febrile children with malignancies is always the more willing choice for pediatric clinicians, the guidelines of vast medical institutions and multitudinous countries to avoid further progression and exacerbation (9–11). In view of the adverse effects from the frequent use of wide-spectrum antibiotics in a high grade, such as dysbiosis throughout multiple systems, opportunistic infections, and expensive treatment payment, pathogen-targeted antibiotic therapy based on validated pathogen-detected results become increasingly urgent for global clinicians. In contrast to the limitations of a conventional culture-dependent technique, the lower rate of positive results, longer test course, and critical requirement for objective samples, emerging 16s RNA tests show advantages unfolding the full view of one individual microbiome (12–14), revealing the microbial changes in infectious loci within a certain group of individuals.

There are limited data regarding the lung microbiome from samples of the pediatric malignancy group, and most of the general information is extrapolated from adult studies. Further studies are needed to establish the optimal approach to the diagnosis and management of children with hematological respiratory infections. Thus, in this study, we sought to characterize the lung microbiome from bronchoalveolar lavage fluid (BALF) samples in a cohort of 16 children with hematological malignancies and moderate-to- severe lower respiratory tract infections (LRTIs) versus 21 LRTI controls with age, gender, weight, and infection severity matched and without any underlying malignancies.

## 2. Methods

### 2.1 Clinical cohort and sample collection

From January 2020 to October 2021, we prospectively enrolled a convenience sample of consecutive malignancy children with moderate-to-severe LRTIs and, at the same time, LRTI children without any underlying diseases as controls, with age, gender, weight, infection severity matched, who all needed bronchoscopy and alveolar lavage in pediatric departments at Qilu Hospital of Shandong University and who had focal findings on a lung CXR or CT scan diagnosed as LRTIs and had bronchoscopy lavage management within the first two days after hospitalization. LRTIs were defined as more than one of the following: new or different cough or sputum production, chest pain, dyspnea, tachypnea, or abnormal auscultatory findings (15). Hematological malignancies were all pathologically diagnosed based on bone marrow cytology smears, lymph node biopsy, and immunophenotyping. All enrolled children were eligible for the indications of the Guidelines of Pediatric Flexible Bronchoscopy in China (2018 version): (1) laryngeal stridor; (2) recurrent or persistent wheeze; (3) local stridor; (4) chronic cough of unknown cause; (5) recurrent respiratory tract infection; (6) suspicious foreign body aspiration; (7) hemoptysis; (8) difficulty in weaning mechanical ventilation; and (9) abnormal imaging results in lungs: (1) dysplasia or malformation in trachea or bronchi; (2) atelectasis; (3) emphysema; (4) mass lesions of lungs; (5) diffuse lesions of lungs; (6) mediastinal emphysema; (7) space-occupying focus of mediastinum or airway; (8) dysplasia of blood vessels, lymphangion, or esophagus; (9) differentiating the diagnosis of lesions in pleural cavity; (10) pathogenic diagnosis and treatment of infections in lungs;(11) thoracic trauma with suspicious airway rupture; (12) interventional therapy with bronchoalveolar lavage; (13) assessment and management of airway in a perioperative period; (14) assistance of endotracheal intubation and gastric intubation; and (15) other conditions for differentiating diagnosis. In addition, all cases underwent bronchoalveolar lavage until they were excluded by contraindications (such as severe cardiopulmonary hypofunction, severe arrythmia, high fever, severe hemoptysis, and severe malnutrition). Exclusion criteria included details as follows: (1) inability to obtain informed consent; (2)predisposing bronchoscopy and alveolar lavage in other hospitals within the same infection medical course; (3) immunodeficiency; (4) chronic corticosteroid use; (5) chronic lung disease; (6) sickle cell disease; (7) congenital heart disease; (8) patients dependent on tracheostomy; and (9) neuromuscular disorders impacting respiration. The study was approved by Qilu Hospital of Shandong University Institutional Review Board (Protocol KYLL-2020(KS)-211), and written informed consent was provided by all participants’ legal guardians. Upon enrollment, we collected BALF for the study of the lung microbiome with simultaneous blood samples for the quantification of the host inflammatory response and immune state.

### 2.2 Laboratory analyses

We extracted bacterial DNA directly from frozen BALF samples and amplified the V3–V4 hypervariable region of the bacterial 16S ribosomal RNA (rRNA) for sequencing on the Illumina Novaseq platform. Simultaneously, we performed quantitative PCR (qPCR) of the V3–V4 to obtain number of 16S rRNA gene copies per sample (surrogate for bacterial load). The default set of criteria was used to remove low-quality and chimeric reads. The remaining reads were subject to a close reference operational taxonomic unit (OTU) picking (97% identity cutoff). As for plasma biomarkers, we performed routine tests (white blood cells, neutrophils, red blood cell count, hemoglobin, platelets, alanine transaminase, etc.), inflammatory biomarkers (CRP, PCT, et al.) and correlated measurements. The WBC count, ANC, and ESR assays were performed on the CELL-DYN Sapphire (Abbott Diagnostics, Lake Forest, IL). CRP assays were performed on the Dimension Vista 1500 (Siemens Medical Solutions USA, Inc, Malvern, PA, USA) with a functional sensitivity of 0.29 mg/dl. Biomarkers were concentrations in blood measured at the Center for Clinical Research of Qilu Hospital of Shandong University.

### 2.3 Sequencing data quality controls

First, Trimmomatic (16) (version 0.33) was used to filter the quality of the raw data, then use Cutadapt (version 1.9.1) to identify and remove primer sequences, and then use FLASH (version 1.2.11) to splice the paired-end reads, with chimeras were removed (UCHIME, version 8.1), resulting in high-quality sequences for subsequent analysis. The original off-machine subreads were corrected to obtain CCS (Circular Consensus Sequencing) sequences (SMRT Link, version 8.0), and then using the Lima (v1.7.0) software, the CCS sequences of different samples were identified by barcode sequences and chimeras were removed to obtain high-quality CCS sequence.

### 2.4 Amplicon generation

The diluted genomic DNA was used as a template; specific primers with Barcode were used according to the selection of the sequencing region; Phusion^®^ High-Fidelity PCR Master Mix with GC Buffer was used. The PCR was performed using efficient and high-fidelity enzymes to ensure amplification efficiency and accuracy. The primer corresponding area is as follows: 16S V3+V4 338F 5’- ACTCCTACGGGAGGCAGCA-3’, 806R 5’- GGACTACHVGGGTWTCTAAT-3’.

### 2.5 PCR product mixing and purification

The samples were mixed at the same concentration according to the concentration of the PCR product, thoroughly mixed, and the PCR product was detected by 2% agarose gel electrophoresis, and GeneJET gel (Thermo Scientific) was used. The product was recovered.

### 2.6 Data analysis

#### 2.6.1 Sequencing data processing

The raw data obtained by the Illumina MiSeq/HiSeq sequencing platform have some low-quality data that will interfere with the final result. Therefore, it is necessary to preprocess the offline data before further analysis. The specific processing steps are as follows: pata splitting, PE reads stitching, Tags filter and Tags to chimera sequences. The PE read splicing is performed with the application of Usearch v10.0 to split the data for the reading of each sample. Raw Tags are also the stitching sequences obtained.

#### 2.6.2 Operational taxonomic unit cluster and species annotation

All of the Effective Tags sequences of all samples were clustered using Usearch v10.0 software (17), providing clustering with 97% consensus sequences to become OTU results, which purpose is to study the compositional diversity information of the species of the sample. A sequence in the same OTU is considered to be sequence-derived from one of the same taxon as the hypothetical taxon. When Usearch constructs OTUs, it selects the sequence with the highest frequency according to its algorithm principles and uses these RDP Classifier and GreenGene database for species annotation analysis to study the phylogenetic relationship between OTUs and uses KRONA for species identification. The results of the annotations are visualized. Based on the species annotation, the number of sequences for each sample at each classification level is calculated, and the sequence of species constitutes a histogram.

To facilitate a further study of the phylogenetic relationships of OTUs and the structural differences of major flora between different samples (groups), phylogenetic relationship data for the first 10 genera of OTUs corresponding to the maximum relative abundance were selected and combined with each OTU. With the relative abundance and species annotation confidence for representative sequences, the results of the integration can visualize the diversity of the species composition of the study. According to the type labeling and abundance information of all samples of the genus level, select the top 35 abundance genera and their abundance information in each sample to draw a heat map, and collect clusters from the difference between the classification information and the sample to identify Focus on more species or samples in the study sample. Select the phylogenetic relationship data of the OTUs corresponding to the top 10 relatives of the largest relative abundance and the relative abundance information of their corresponding OTUs to achieve a vertical clustering of samples at the OTU level to examine the differences between different samples or Similarity.

#### 2.6.3 α diversity

α Diversity is used to analyze community diversity within a sample and includes three indicators: dilution curve, species richness, and community diversity (18). The sample complexity index was calculated and plotted using Qiime2 software. The rarefaction curve is used to indicate whether the amount of sequencing data of the sample is reasonable and indirectly reflects the richness of the substance in the sample. It is a curve obtained by randomly extracting a certain amount of sequencing data from a sample to calculate the number of species they represent based on the number of species and the amount of data. In the dilution curve, when the curve tends to be flat, it means that more data will only produce a small amount of new OTUs, indicating that the amount of sequencing data is reasonable.

#### 2.6.4 β diversity

Principal component analysis (PCA) is a method for the dimensionality reduction of multidimensional data and the most important elements and structures in the data by applying variance decomposition (19). It is applied to reduce the dimension of the original variables using the QIIME2 software package. It can reflect the difference of multidimensional data on the two-dimensional coordinate map, and the method of selecting the two coordinate axes that can reflect the difference between samples is selected from the PCA results. The closer the sample is in the PCA plot, the more similar its community composition. UPGMA (unweighted pair-group method with arithmetic mean) is a commonly used cluster analysis method in environmental biology. It requires a transformation from the distance matrix to a new set of orthogonal axes, where the maximum variation factor is represented by the first principal coordinate, the second maximum is represented by the second primary coordinate, and so on. UPGMA clustering is a hierarchical clustering method that uses average links and can be used to interpret the distance matrix.

#### 2.6.5 Function prediction analyses

PICRUSt2 (20) is a computational method that uses marker gene data and a reference genome database to predict the functional composition of environmental microbes, which is based on IMG microbial genome data to predict the functional potential of microbial communities during phylogeny through phylogenetic and functional correlations. Its working principle is summarized in **Supplementary Figure 1**.

BugBase is a biological-level coverage that predicts functional pathways within the complex microbiome and Methods for biologically interpretable phenotypes. BugBase first normalizes OTUs by the predicted 16S copy number and then predicts microbial phenotypes using the provided precomputed files. First, for each sample in the biological dataset, the relative abundance of the trait across the full range of coverage thresholds is estimated (0–1 in 0.01 increments). Then, BugBase selects the coverage threshold with the highest variance among all samples for each feature in the user data. After thresholds are set, BugBase generates the final organism-level trait prediction table, which contains the predicted relative abundance of the trait for each sample.

#### 2.6.6 Data processing and statistical analyses

From the derived 16S sequences, we applied a custom pipeline for OTU classification and performed analyses at phylum, class, order, family, genus, and species levels, respectively. Statistical analysis groups were compared using the Fisher exact or chi-square tests for categorical data or the Wilcoxon rank sum test for continuous data. A general linear model (GLM) was constructed between demographic and baseline clinical variables and the temporal variability of α and β diversity among subjects. The correlation analysis between two indicators was done using Spearman correlation analysis. The false discovery rate (FDR) method was used to adjust P-values for multiple testing wherever applicable (21). All tests were two tailed and a P-value <0.05 was considered to be statistically significant. IBM SPSS statistics (version 21.0; International Business Machine Corp.) was used. The ecologic analyses of α diversity (Shannon index) and β diversity (Manhattan distances with permutational ANOVA [Permanova] at 1,000 permutations) were conducted using the R vegan package and visualized with principal-coordinate analysis plots.

## 3. Results

### 3.1 Cohort description

A total of 16 children with malignancies and moderate-to-severe LRTIs (median age, 4.6 years, 62.5% male) contributed to the observation group (75% acute lymphoblastic leukemia, 12.5% acute myelogenous leukemia, 6.25% B-cell lymphoblastic lymphoma, 6.25% Burkitt’s lymphoma), in addition to the control group of 21 children with LRTIs only (median age, 3.9 years, 42.9% male) (**Table 1**) (the chemotherapy information of children with malignancy and LRTIs is listed in **Table 2**). For both groups, bronchoscopy was strongly needed for moderate-to-severe LRTIs. Comparison samples including blood and BALF from the samples of 16 observation-group objects and 21 control-group objects were used in our experiment pipelines.

**Table 1:** Comparison of the demographics, clinical course, features, and outcome of lower respiratory tract infection (LRTI) children with/without hematological malignancies.

Characteristics	Children with hematological malignancies and sLRTI (N = 16)		Children with sLRTI only (N = 21)	P-value*
Age, median (IQR), years	4.6 (3.5–7.7)		3.9 (1.7–8.1)	0.471
Weight, median (IQR), kg	16.8 (14.0–24.0)		19.0 (10.4–29.5)	0.923
Height, median (IQR),cm	105.5 (98.0–127.3)		105.0 (85.0–135.0)	0.581
Males, n (%)	10 (62.5)		9 (42.9)	0.236
Malignancy subtype, n (%)	ALL	12 (75.0)	–	–
	AML	2 (12.5)	–	–
	B-LBL	1 (6.25)	–	–
	BL	1 (6.25)	–	–
Course of lower respiratory tract infection before hospitalization, median (IQR), d	5 (1–15)		4 (3–11)	0.805
Course of antibiotics before hospitalization, median (IQR), d	3 (1–14)		7 (0–18)	0.916
Course of entire lower respiratory tract infection, median (IQR), d	31 (19–38)		13 (11–20)	**0.001**
WBC, median (IQR), ×10^9^/L	3.30 (2.13–21.49)		7.55 (5.26–13.17)	0.456
ANC, median (IQR), ×10^9^/L	1.42 (0.58–5.92)		2.79 (1.95–6.75)	0.059
RBC, median (IQR), ×10^12^/L	2.67 (2.13–3.52)		4.61 (4.21–4.91)	**0.000**
Hb, median (IQR), g/L	84 (64–113)		125 (119–132)	**0.000**
PLT, median (IQR), ×10^9^/L	244 (35–310)		328 (313–416)	**0.000**
CRP, median (IQR), mg/L	15.29 (1.91–81.47)		2.06 (0.25–14.4)	**0.044**
ALT, median (IQR), g/L	15 (10–45)		13 (9–22)	0.421
LDH, median (IQR), g/l	303 (237–356)		335 (292–412)	0.217
Positive BALF cultures, n (%)	1 (6.3)		4 (19.0)	0.259
Positive cultures other than BALF, n (%)	4 (25.0)		0 (0)	**0.015**
FEV1/FVC, %, median (IQR), (n)^**^	106.6 (100.5–115.1), (7)		108.5 (98.6-113.1), (9)	0.965
FEV1, %, median (IQR), (n)^**^	90.2 (78.7–102.6), (7)		85.7 (74.9-94.5), (9)	0.480
MEF75, %, median (IQR), (n)^**^	73.9 (60.9–83.8), (7)		69.0 (64.4–95.8), (9)	0.895
MEF50, %, median (IQR), (n)^**^	68.4 (64.3–99.8), (7)		70.0 (52.3–100.8), (9)	0.965
MEF25, %, median (IQR), (n)^**^	71.5 ()58.3–98.8), (7)		73.5 (52.7–95.0), (9)	0.895
MEF75/25, %, median (IQR), (n)^**^	70.2 (64.3–101.7), (7)		71.0 (60.3–98.5), (9)	0.965
FVC, ml, median (IQR), (n)^**^	90.1 (79.0–95.0), (7)		80.0 (66.5–93.3), (9)	0.402
VT/kg, ml/kg, median (IQR), (n)^**^	8.9 (5.8–11.2), (9)		12.4 (11.1–14.4), (12)	**0.002**
tPTEF/tE, %, median (IQR), (n)^**^	18.3 (15.7–36.6), (9)		19.7 (14.0–36.9), (12)	0.865
VPEF/VE, %, median (IQR), (n)^**^	20.4 (18.9–37.6), (9)		24.2 (19.6–33.6), (12)	0.777
Hospitalization course, median, d	17.5 (4–52)		7 (4–14)	**0.000**
Death during sLRTI, n (%)	1 (6.3)		0 (0)	0.245

sLRTI, severe lower respiratory tract infection; ALL, acute lymphoblastic leukemia; AML, acute myelogenous leukemia; B-LBL, B-cell lymphoblastic lymphoma; BL, Burkitt’s lymphoma; WBC, white blood cell count; ANC, absolute neutrophil count; RBC, red blood cell count; Hb, hemoglobin; PLT, platelet; CRP, C reactive protein; ALT, alanine transaminase; LDH, lactate dehydrogenase; BALF, bronchoalveolar lavage fluid.

*Statistically significant p-values(p < 0.05) are shown in bold.

^**^Children under 6 years old were only given tidal breath pulmonary function. Children older than 6 years were given a pulmonary function test.

**Table 2:** Chemotherapy information of children with hematological malignancies and LRTIs.

Malignancies subtype (n)	Disease group		Malignancy course/m	Present chemotherapy phase when enrolled	
ALL (12)	Mediate risk	5 (42%)	3 (0.55–20)^*^	Induction phase	6 (50%)
				Consolidation phase	2 (16.7%)
	Low risk	7 (58%)		Maintenance phase	4 (33.4%)
AML (2)	Low risk	2 (100%)	1/1.5	Induction phase	2 (100%)
B-LBL (1)	Stage III		15	Consolidation phase	
BL (1)	Stage IV		10	3 days after the end of chemotherapy	

^*^Median (IQR).

With respect to the pathogen of LRTIs in the observation group, there were two bacterial infection cases (one case of penicillin-sensitive *Staphylococcus aureus* infection defined by a BALF culture, one case of *Haemophilus influenzae* infection defined by BALF PCR), two mycoplasma pneumoniae-infected cases defined by BALF PCR, two viral infection cases (one case of adenovirus infection and one case of coronavirus infection defined both by BALF PCR), and two suspicious fungal infection cases defined by positive serum (1,3)-beta-D-glucan test (G test), the galactomannan test (GM test) plus a high level of the BALF GM test (>1.50 ODI) (22) without other conventional diagnostic bases of fungal infection, with eight cases of no pathogens tested. In the control group, there were seven bacterial infection cases [one case of *Branhamella catarrhalis* infection defined by a BALF culture, two cases of *H. influenzae* infection defined by a BALF culture, and four cases of *S. pneumoniae* infection defined by a BALF culture (n=1) and BALF PCR (n=3)], five mycoplasma pneumoniae-infected cases defined by BALF PCR, and two viral infection cases (one case of parainfluenza virus infection and one case of herpes virus infection defined both by BALF PCR), with seven cases of no pathogens tested.

As for the pulmonary function tests, children under 6 years old were only given tidal breath pulmonary function (e.g., VT/kg, tPTEF/tE, and VPEF/VE). Children older than 6 years were given a pulmonary function test (e.g., FEV1/FVC, FEV1, MEF75, MEF50, MEF25, MEF75/25, and FVC). Among the 16 children with malignancy and LRTIs, there were 7 children who were given a routine pulmonary function test and 9 children who were given a tidal breath pulmonary function test. Among the control group, there were 9 children who were given a routine pulmonary function test and 12 children who were given a tidal breath pulmonary function test. With regard to the phenotypes in pulmonary function tests, in children with malignancy and LRTIs, there were two mediate obstructive ventilation dysfunction, two mediate–severe obstructive ventilation dysfunction, three mild restrictive ventilation dysfunction, and four mediate restrictive ventilation dysfunction, while there were other five children in this group with common pulmonary function. Within the control group, there were three mild obstructive ventilation dysfunction, three mediate obstructive ventilation dysfunction, two severe obstructive ventilation dysfunction, two mild restrictive ventilation dysfunction, and two severe obstructive plus mild restrictive ventilation dysfunction, while for the rest nine children in this group, with common pulmonary function.

About the phenotypes of CT scan, there were 11 pneumonia and 5 bronchopneumonia in children with malignancy and LRTI group, with 2 pneumonia cases complicated with pleural effusions and 1 pneumonia case complicated with pulmonary cavitation. Furthermore, within this group, there were 1 single left lesion, 4 single right lesions, and 11 bilateral lesions. Meanwhile, in the control group, there were 9 pneumonia and 12 bronchopneumonia in children, with 1 pneumonia case complicated with pleural effusions. Within the control group, there were 5 single left lesions, 2 single right lesions, and 14 bilateral lesions.

### 3.2 Description of the clinical features in lower respiratory tract infection children with/without hematological malignancies

There were no significant differences noted in the age, weight, height, gender, predisposition of antibiotics before hospitalization, white blood cell count, absolute neutrophil count, C-reactive protein, alanine transaminase, lactate dehydrogenase, positive BALF cultures, FEV1/FVC, FEV1, MEF75, MEF50, MEF25, MEF75/25, FVC, tPTEF/tE, VPEF/VE, and death during LRTIs between the observation group and the control group (**Table 1**). Meanwhile, LRTI children with hematological malignancies revealed to have less red blood cell count (median, 2.67 ×10^12^/L *vs*. 4.61 ×10^12^/L, p<0.001), lower hemoglobin level (median, 84g/L *vs*. 125g/L,p<0.001), less platelet count(median, 244 ×10^9^/L *vs*. 328 ×10^9^/L, p<0.001), higher incidence of positive cultures of non-respiratory samples (25% *vs*. 0%, p=0.015), lower VT/kg(median, 8.9 *vs*. 12.4, ml/kg, p=0.002, <0.01), and longer entire lower respiratory tract infection course (median, 31 *vs*. 13,p=0.001, <0.01) and hospitalization course (median, 17.5 *vs*. 7, p<0.001) than those with LRTIs but without hematological malignancies (**Table 1**).

### 3.3 Lung microbiome communities in lower respiratory tract infection children with or without hematological malignancies

**Figure 1:**
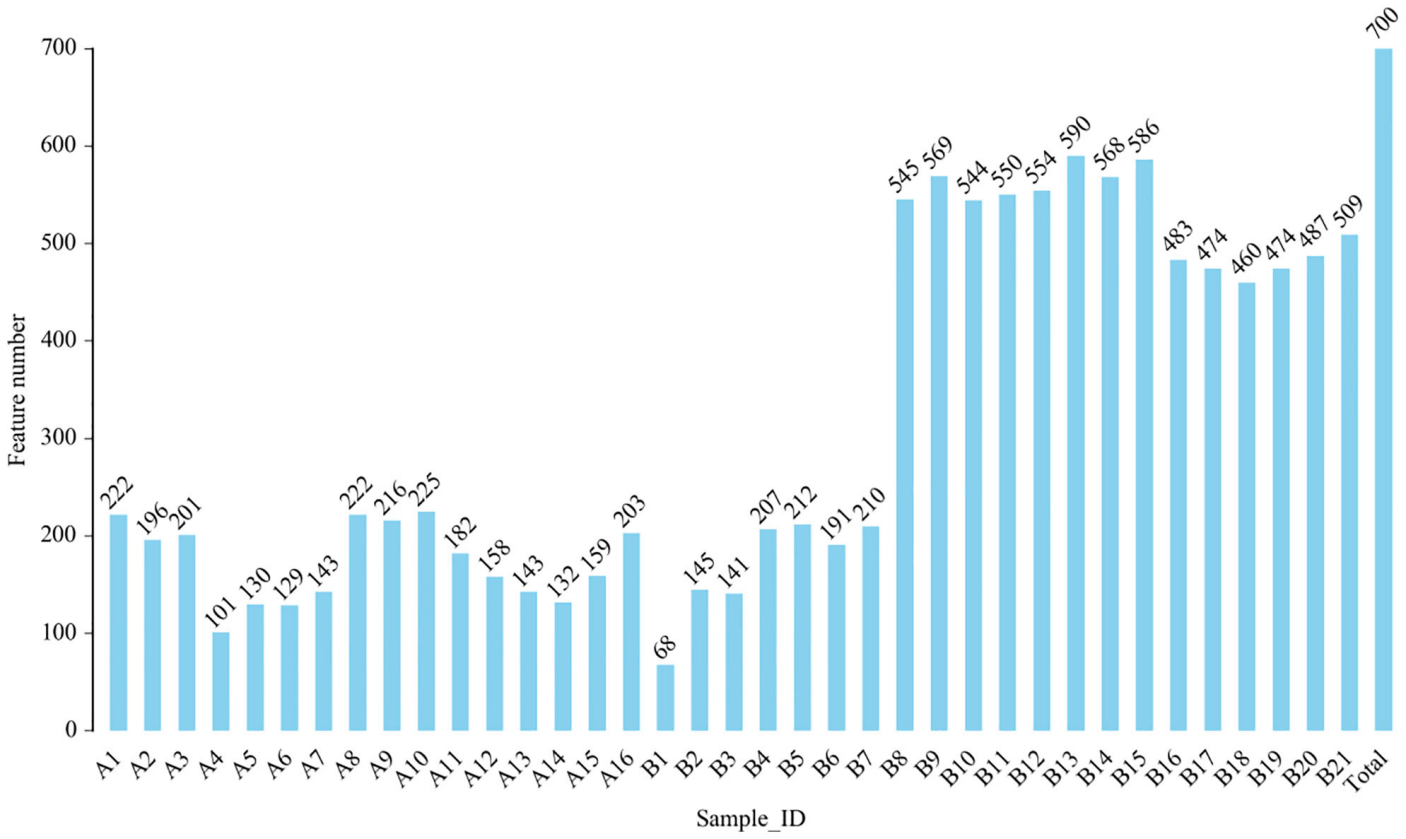
Comparison of operational taxonomic units (OTUs) in bronchoalveolar lavage fluid (BALF) from lower respiratory tract infection (LRTI) children with/without hematological malignancies. P = 0.001, < 0.01, feature number of group **A(A1-16)** was obviously lower than that of group **B(B1-21)**. A1–16 stand for the BALF samples of LRTI children with hematological malignancies, while B1–21 stand for the samples of LRTI children without malignancies.

**Figure 2:**
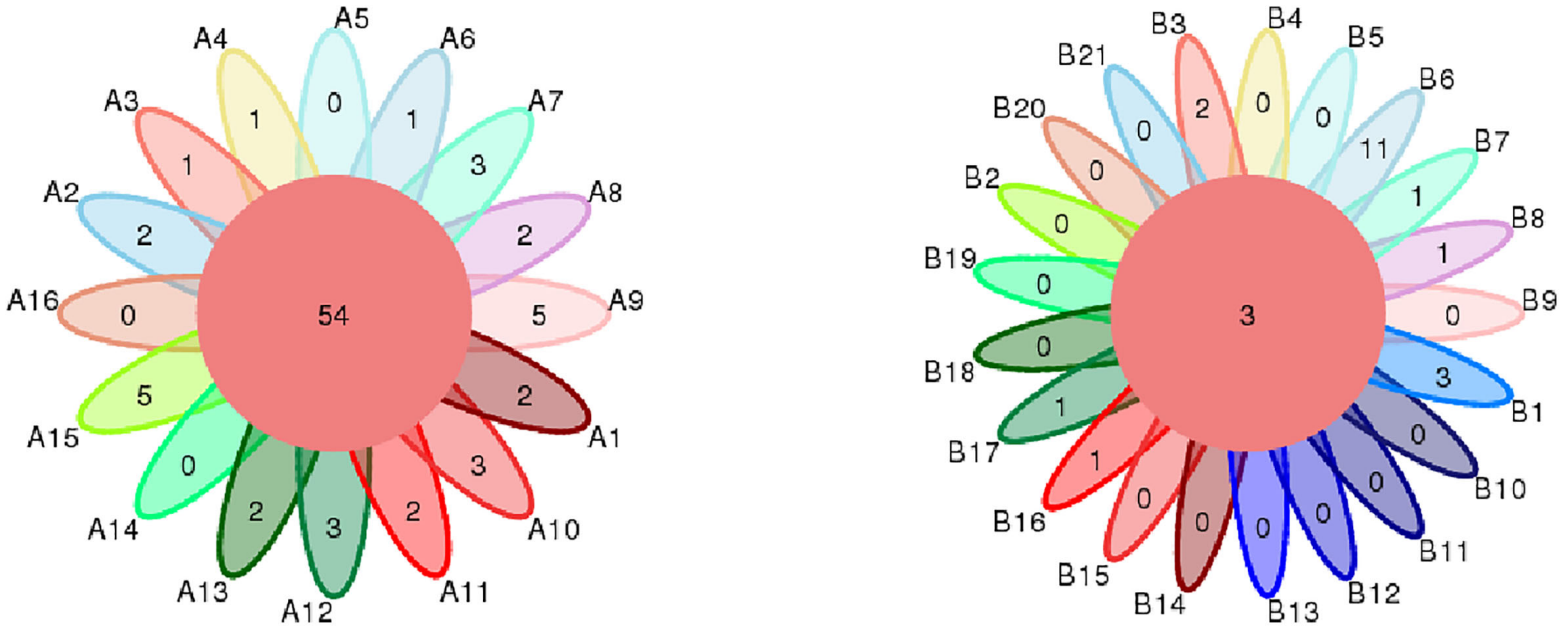
Comparison of mutual OTUs of samples from LRTI children with/without hematological malignancies. The left figure reflects BALF samples of LRTI children with hematological malignancies (Group A); the right figure reflects BALF samples of LRTI children without hematological malignancies (Group B). Mutual OTUs (the core of flower) of group **A** were more than that of group **B**.

**Figure 3:**
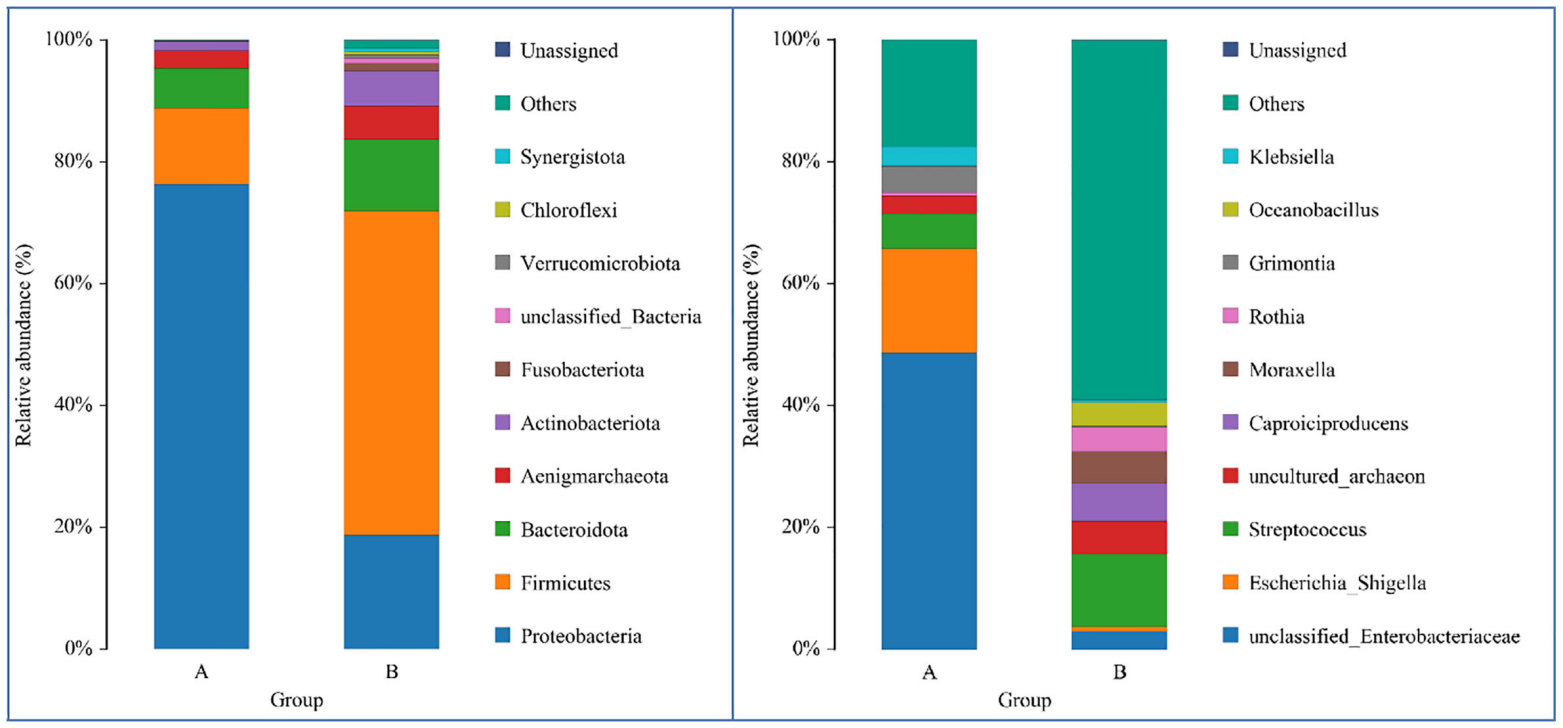
Comparison of LRTI children with/without hematological malignancies at the phylum/genus level. The bar chart on the left was the comparison at the phylum level, while the bars on the right were at the genus level. Bar A reflects BALF samples of LRTI children with hematological malignancies; Bar B reflects BALF samples of LRTI children without hematological malignancies.

From DNA sequences of the V3 and V4 hypervariable regions of the 16S rRNA gene, a total of 700 OTUs were clustered by the identity of 97% cutoff after rarefaction to make species annotation. There were 79,854–112,096 raw reads, 73,966-104,672 clean reads and 54,778–95,159 effective reads analyzed of each sample. The mean Q30(%) was 95.96% and the mean Q20(%) was 98.93%. The samples of bronchoalveolar lavage fluid (BALF) from LRTI children with hematological malignancies had a much lower number of 16S reads than samples from control group(p=0.001, <0.01) (**Figure 1**), whereas the mutual OTUs of samples from the observation group were much more than those from the control group (**Figure 2**). The microbiome composition between the two groups was significantly different. Inconsistent with the previous studies of the lung microbiome, the vast majority of OTUs in LRTI children with hematological malignancies belonged to *Proteobacteria* (76%), *Firmicutes* (12%), *Bacteroidota* (7%), and *Actinobacteriota* (2%) at the phylum level, while the most abundant in LRTI children without hematological malignancies belonged to *Firmicutes* (53%), *Proteobacteria* (19%), *Bacteroidota* (12%), and *Actinobacteriota* (6%), similar to other studies. At the genus level, the top two in LRTI children with hematological malignancies belonged to *unclassified_Enterobacteriaceae* (49%) and *Escherichia_Shigella* (17%), whereas the vast two in LRTI children without hematological malignancies were *Streptococcus* (12%) and C*aproiciproducens* (6%) (**Figure 3**). Other common genera, such as *Moraxella* (5%)*, Bacillus*(4%)*, Oceanobacillus* (4%), and *Rothia*(4%), were among the most abundant genera of BALF identified in children without malignancies, while in children with malignancies, they were *Streptococcus* (6%)*, Grimontia* (4%) and *Klebsiella*(3%), *Bacteroides* (3%), and *Parabacteroides* (3%).

### 3.4 α diversity of lung microbiome in lower respiratory tract infection children with or without hematological malignancies

**Figure 4:**
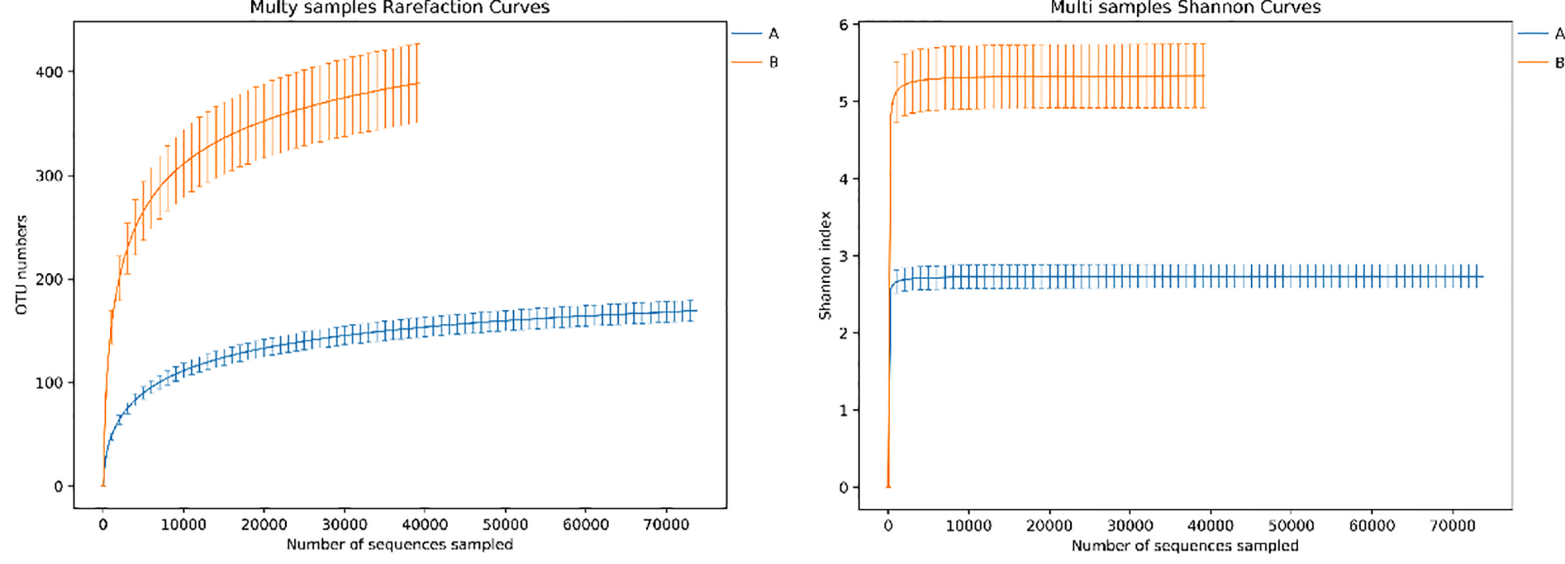
Rarefaction curve and Shannon index curve between samples of LRTI children with/without hematological malignancies. Curve A reflects BALF samples of LRTI children with hematological malignancies; Curve B reflects BALF samples of LRTI children without hematological malignancies.

**Figure 5:**
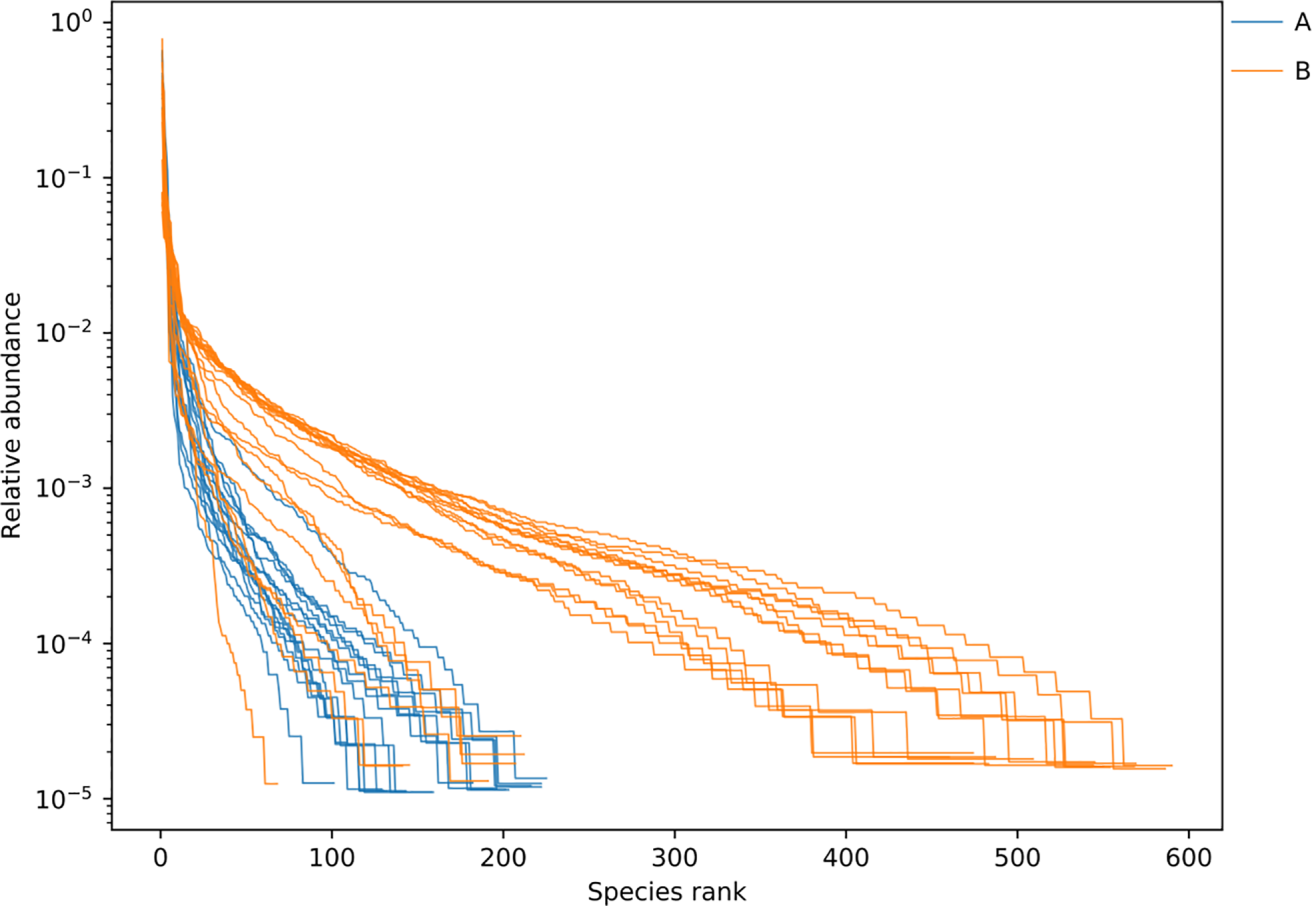
Rank abundance curve between samples of LRTI children with/without hematological malignancies. Curve A reflects BALF samples of LRTI children with hematological malignancies; Curve B reflects BALF samples of LRTI children without hematological malignancies. Width of each curve means the species richness of certain samples; smoothness means the species evenness of certain samples.

Overall, the α (within‐subject) diversity between LRTI children with or without hematological malignancies was significantly different (ACE p=0.000, Shannon p=0.000, Chao1 p=0.000, and Simpson p=0.000, p<0.01) (**Table 3**). LRTI children with hematological malignancies were found with a much lower number of OTUs and a smaller Shannon index than that of the control group (**Figure 4**). The richness and evenness of species from the BALF of LRTI children with hematological malignancies decreased significantly than those of the control group (**Figure 5**). For the comparison of abundances in common species at the phylum or genus levels, respectively, between two groups, obvious differences were found in *Proteobacteria, Firmicutes*, *Actinobacteriota*, and *Bacteroidota* at the phylum level, in addition with significant differences at the genus level, in *Escherichia_Shigella*, *unclassified_Enterobacteriaceae, Streptococus, Rothia, Klebsiella*, *Bacteroides*, and *Parabacteroides* (**Table 2**). We performed the correlation analysis of α diversity (Shannon) across different species at the phylum and genus levels, respectively. At the phylum level, *Proteobacteria* was obviously negatively associated with *Firmicutes* (r=-0.909, p=0.000, <0.01) and *Bacteroidota* (r=-0.518, p=0.001, <0.01); in addition to that, *Firmicutes* and *Bacteroidota* were significantly positively correlated with each other (r=0.456, p=0.005, <0.01). At the genus level, *Escherichia_Shigella* was significantly positively associated with *unclassified_Enterobacteriaceae* (r=792, p=0.000, <0.01)*, Klebsiella* (r=0.458, p=0.004, <0.01), and *Parabacteroides* (r=0.354, p=0.032, <0.05), while *unclassified_Enterobacteriaceae* was obviously positively correlated with *Klebsiella* (r=0.415, p=0.011, <0.05). In addition, *Klebsiella* versus *Parabacteroides* (r=0.624, p=0.000, <0.01) and *Streptococcus* versus *Rothia* (r=0.478, p=0.003, <0.01) both had significantly positive correlation.

**Table 3:** Comparison of α diversity between LRTI children with/without hematological malignancies.

α diversity		Sum of ranks		P- value*
		Children with malignancy and sLRTI (N = 20)	Children with sLRTI only (N = 21)	
ACE		183	520	**0.000**
Chao1		182	521	**0.000**
Simpson^*^		184	519	**0.000**
Shannon index		183	520	**0.000**
Abundance (phylum level)	*Proteobacteria*	459	244	**0.000**
	*Firmicutes*	156	547	**0.000**
	*Actinobacteriota*	206	497	**0.002**
	*Bacteriodota*	230	473	**0.023**
Abundance (genus level)	*Escherichia Shigella*	472	231	**0.000**
	*unclassified Enterobacterioriaceae*	468	235	**0.000**
	*Streptococcus*	289	414	0.660
	*Rothia*	293	410	0.751
	*Klebsiella*	432	271	**0.000**
	*Bacteroides*	314	389	0.774
	*Parabacteroides*	404	299	**0.002**

*Comparison of the Simpson index in two groups were eligible for Student’s t-test (p = 0.000, < 0.05), equal with the p-value by the Wilcoxon rank sum test.

Statistically significant p-values (p < 0.05) are shown in bold.

### 3.5 β diversity of lung microbiome in lower respiratory tract infection children with or without hematological malignancies

**Figure 6:**
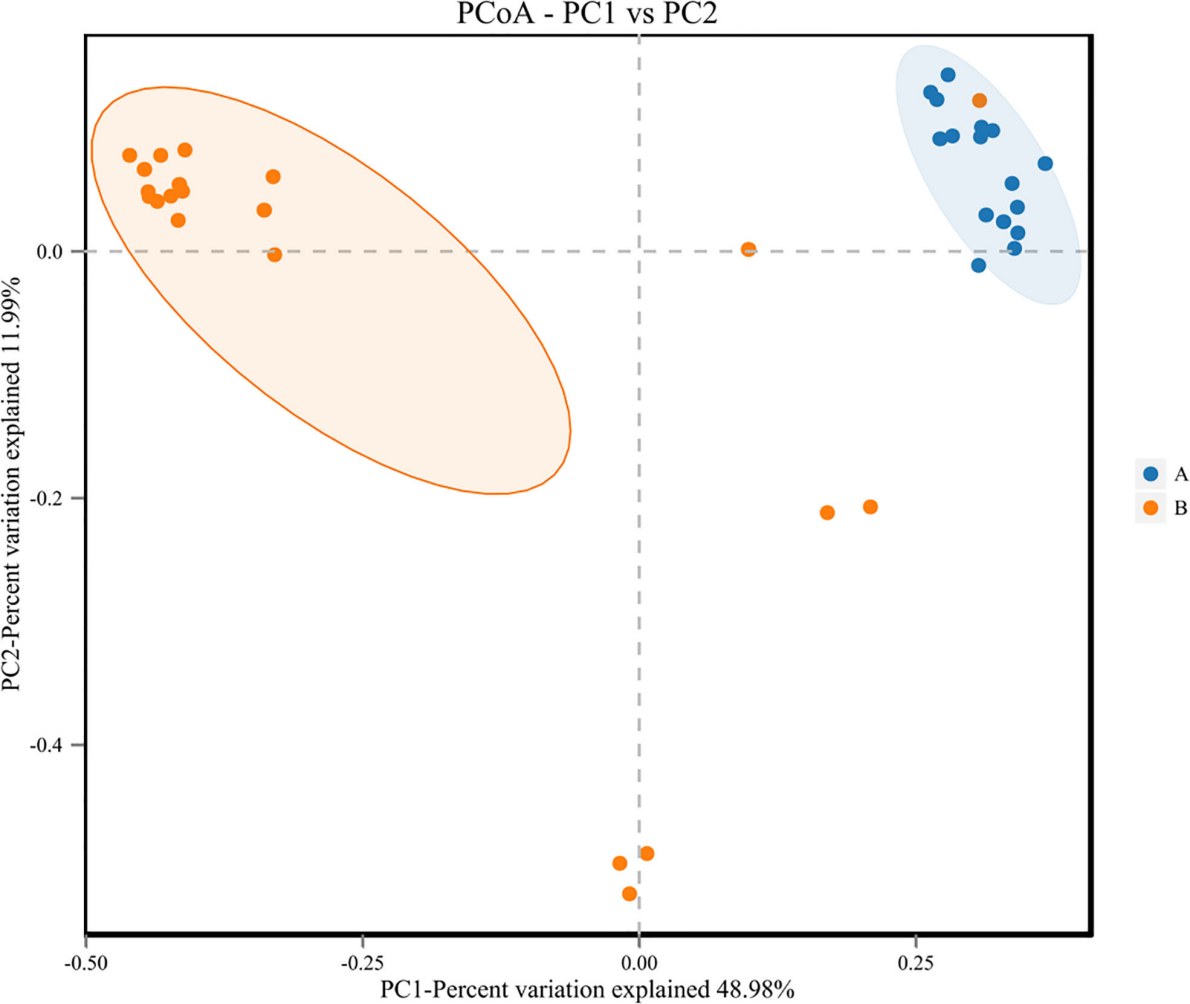
Principal Co-ordinates Analysis (PCoA) of samples of LRTI children with/without hematological malignancies. Dots A reflect BALF samples ofLRTI children with hematological malignancies; Dots B reflect BALF samples of LRTI children without hematological malignancies.

**Figure 7:**
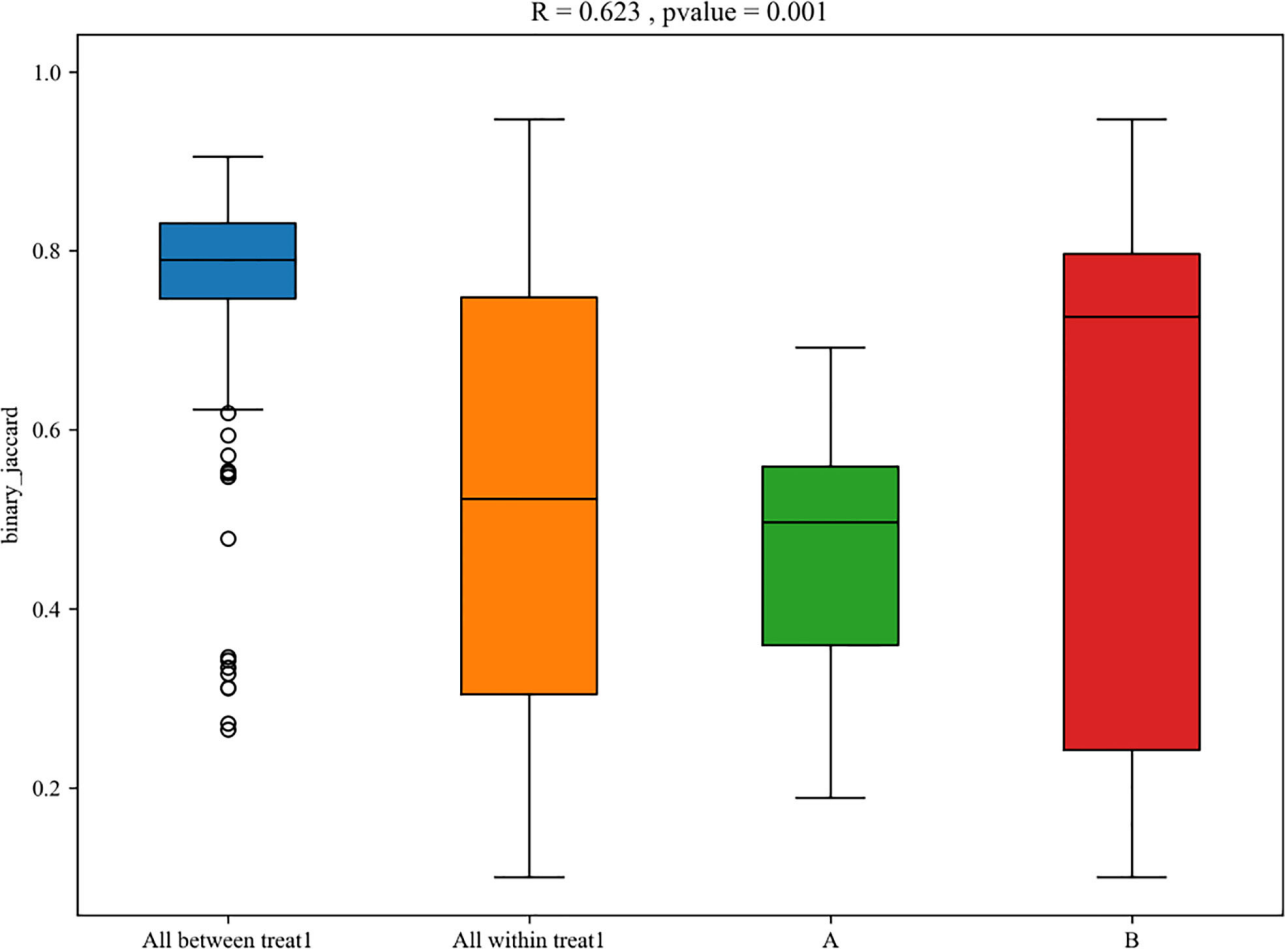
Box plot of PERMANOVA between samples of LRTI children with/without hematological malignancies. A mean BALF samples ofLRTI children with hematological malignancies; B mean BALF samples of LRTI children without hematological malignancies. Treat 1 means the differences(the basis of maignancies and the correlated conditions) between the two groups.

**Figure 8:**
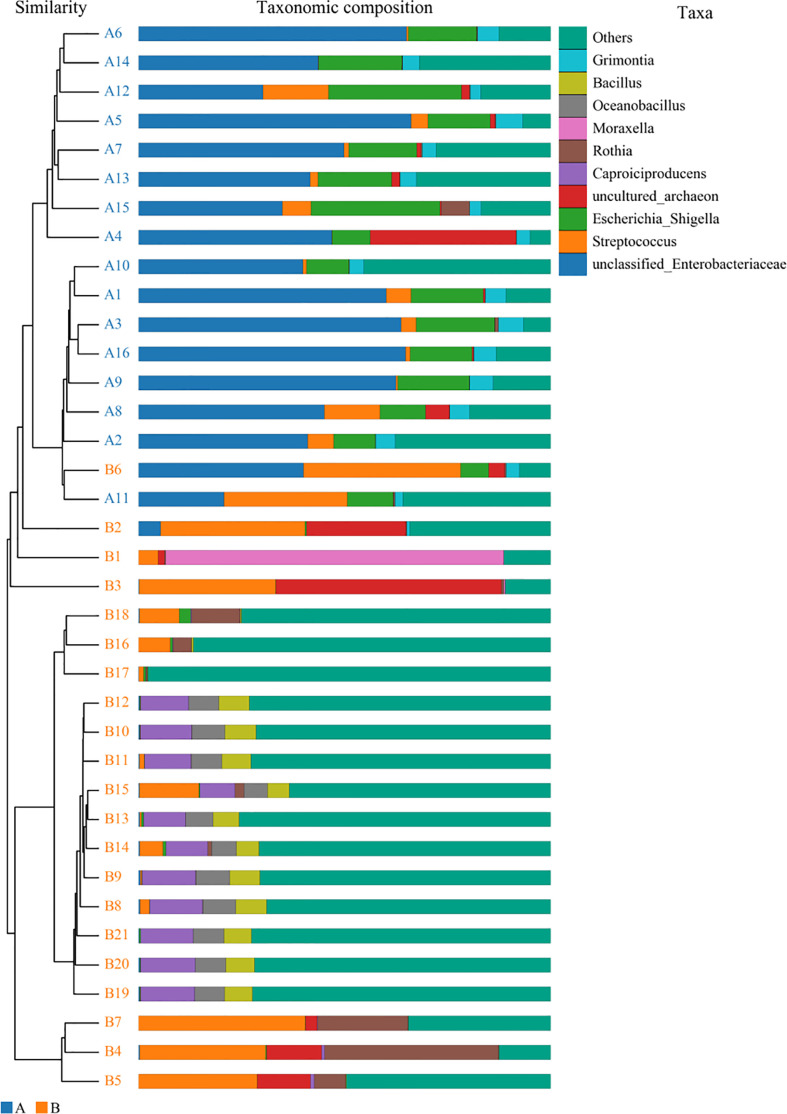
Unweighted pair-group method with arithmetic mean analysis. A1–16 mean the BALF samples of LRTI children with hematological malignancies, while B1–21 stand for the samples of LRTI children without hematological malignancies.

**Figure 9:**
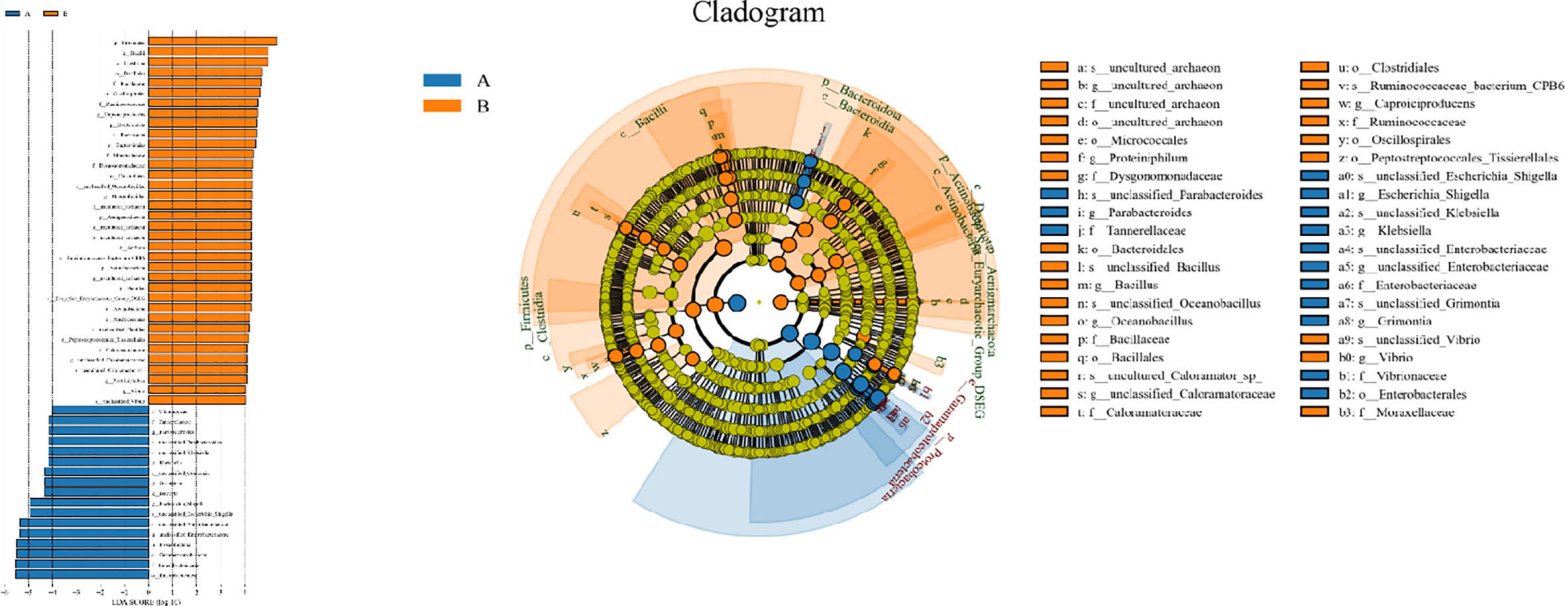
Line discriminant analysis effect size and cladogram. A reflect BALF samples of LRTI children with hematological malignancies; B reflect BALF samples of LRTI children without hematological malignancies.

For the β (between-subject) diversity (Bray–Curtis) analysis, there was a significant difference between two groups (p=0.046, <0.05). The β diversity of BALF samples from LRTI children with hematological malignancies revealed high similarity, while samples from the control group showed higher heterogeneity within the group, with a relatively mutual difference from those from the observation group (**Figure 6**). There were obvious similarities within each group and significant differences between the two groups (**Figure 7**, **8**) (PERMANOVA, R:0.623, p:0.001, p<0.01). As for the linear discriminant analysis effect size (LFeSe) analysis of biomarkers between the two groups, distinct microbial populations were found in bacterial exacerbation among LRTI children with hematological malignancies, with an obvious decreased proportion of *Firmicutes*, *Bacteroidota*, *Aenigmarchaeon*, and *Actinobacteriota* and a significantly increased proportion of *Proteobacteria* at the phylum level. At the genus level, among LRTI children with hematological malignancies, there were obviously decreased proportions of *Caproiciproducens*, *Oceanobacillus*, *Bacillus*, *unclassified_Caloramatoracceae*, *Proteiniphilum*, and *Vibrio* and distinct elevated proportions of *Parabacteroides*, *Klebsiella*, *Grimontia*, *Escherichia_Shigella*, and *unclassified_Enterobacteriaceae* (**Figure 9**).

### 3.6 Function analysis of lung microbiome in lower respiratory tract infection children with or without hematological malignancies

**Figure 10:**
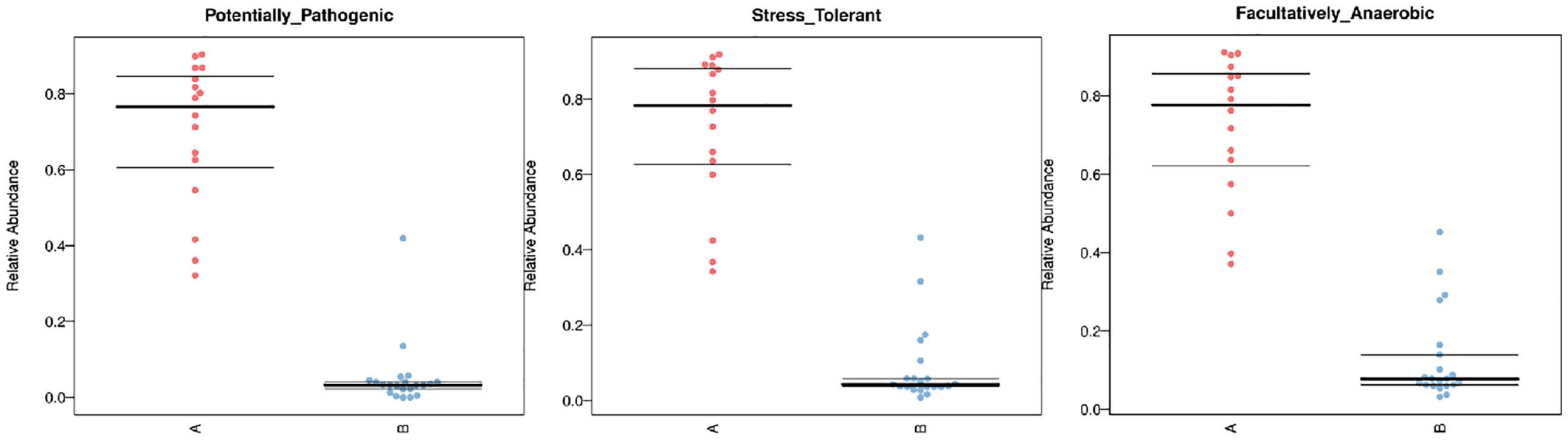
BugBase phenotype prediction. A reflect BALF samples of LRTI children with hematological malignancies; B reflect BALF samples of LRTI children without hematological malignancies.

**Figure 11:**
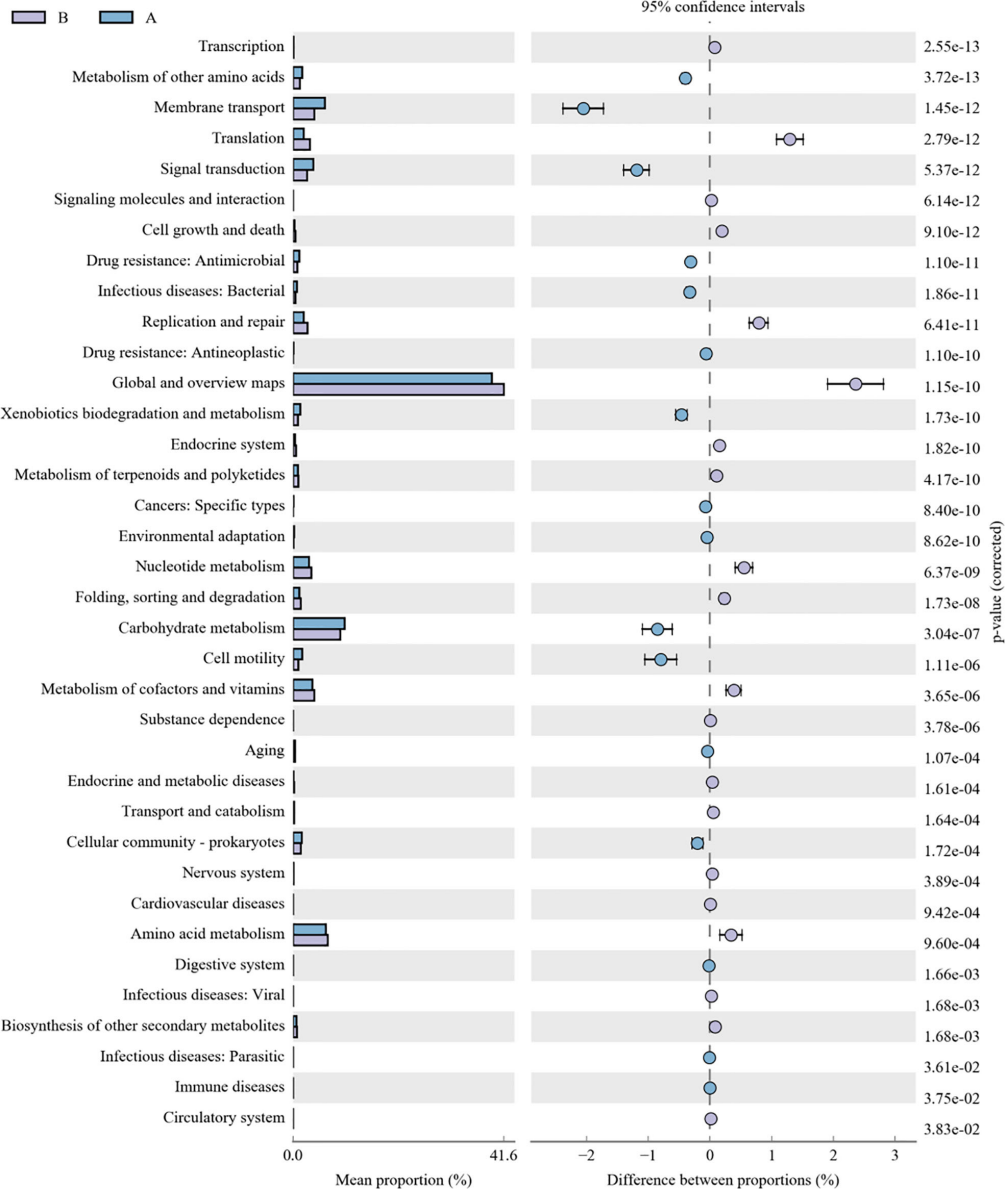
KEGG analysis of LRTI children with/without hematological malignancies. A reflect BALF samples of LRTI children with hematological malignancies; B reflect BALF samples of LRTI children without hematological malignancies.

**Figure 12:**
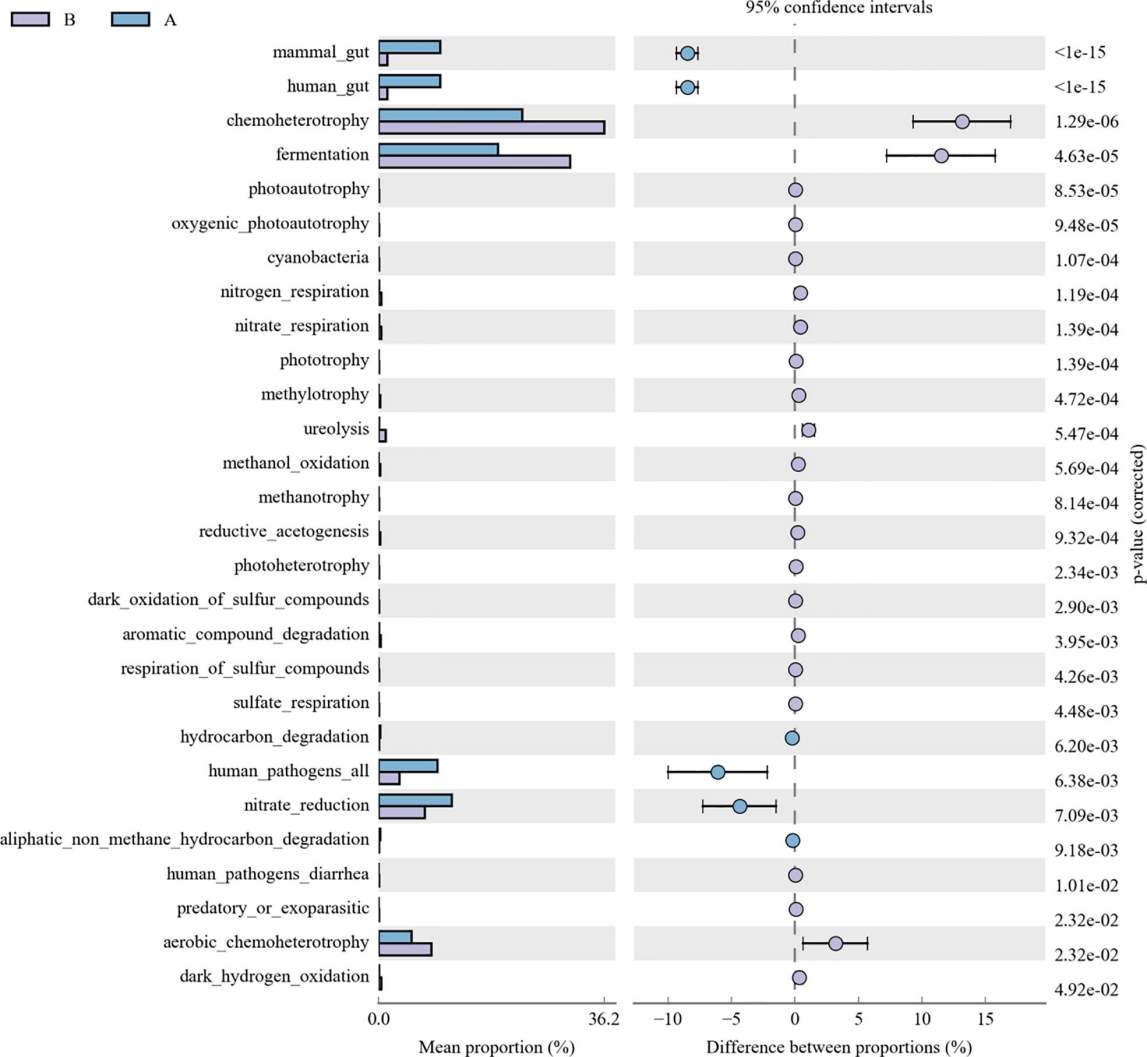
FAPROTAX analysis of LRTI children with/without hematological malignancies. A reflect BALF samples of LRTI children with hematological malignancies; B reflect BALF samples of LRTI children without hematological malignancies.

As for the function analysis, BugBase phenotype prediction, species from LRTI children with hematological malignancies have significantly higher abundance than those from children without malignancies in a potentially pathogenic ability (Mann–Whitney–Wilcoxon test, FDR-corrected p-value: 1.087313e-09), facultatively anaerobic ability (Mann–Whitney–Wilcoxon test, FDR-corrected p-value: 6.213218e-10), and stress tolerance ability (Mann–Whitney–Wilcoxon test, FDR-corrected p-value: 1.087313e-09) (**Figure 10**). For the Kyoto Encyclopedia of Genes and Genomes (KEGG) metabolic pathway analysis between the samples of LRTI children with or without malignancies, there were significant differences in the function of drug resistance: antimicrobial (p-value: 1.01e-11), infectious disease:bacterial (p-value: 1.86e-11), and infectious disease:parasite (p-value: 3.61e-02), all of which were higher in the observation group, whereas there was a mildly decreased function of infectious disease:viral in the lung microbiome of LRTI children with hematological malignancies than that of the control group (p-value: 1.68e-03) (**Figure 11**). For Functional Annotation of Prokaryotic Taxa (FAPROTAX) analysis between these two groups, in terms of mammal_gut (p-value: 1e-15), human_gut (p-value: 1e-15) and human_pathogen_all (p-value: 6.38e-03), LRTI children with hematological malignancies showed a significantly increased microbial function than the control group (**Figure 12**).

### 3.7 Correlation between clinical factors and lung microbiome in lower respiratory tract infection children with or without hematological malignancies

We performed multivariate analysis to identify clinical factors significantly associated with microbial α and β diversity. Among all enrolled clinical factors, alanine transaminase (p=0.012, <0.05) and hemoglobin (p=0.004, <0.05) were significantly correlated with α diversity (Shannon), while the predisposition course of antibiotics before hospitalization (α diversity, p=0.025, <0.05; β diversity, p=0.042, <0.05) revealed an obvious correlation with both α (Shannon) and β (Bray–Curtis dissimilarity) diversity among all samples (**Supplementary Table 1**).

### 3.8 Correlation between clinical factors, lung microbiome in lower respiratory tract infection children with or without hematological malignancies versus hospitalization course

**Figure 13:**
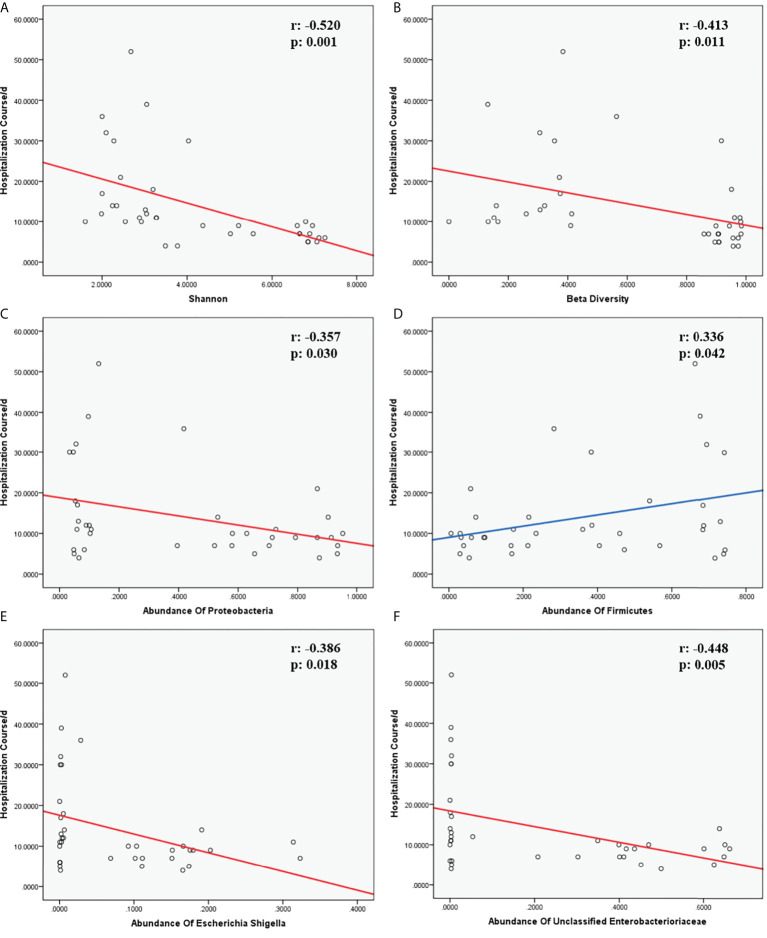
Correlation of hospitalization course and indicators. **(A, B)** were the correlation of α and β diversity and hospitalization course. **(C, D)** were the correlation of species and hospitalization course at the phylum level. **(E, F)** were the correlation of species and hospitalization course at the genus level. The r-value and p-value in Spearman correlation analysis were marked on the top-right corner in each chart. Fitting curves with a negative correlation were in red; fitting curves with a positive correlation were in blue.

Multivariate analysis was performed to identify the correlation between the hospitalization course and the abundances of certain species at the phylum or genus levels, respectively, revealing that *Streptococcus* is correlated with the hospitalization course (R^2 =^ 0.335, p=0.036, <0.05). In addition, we performed multivariate analysis to investigate the association of clinical factors and the hospitalization course, establishing that absolute neutrophil count (p=0.038, <0.05), alanine transaminase (p=0.007, <0.01), hemoglobin (p=0.008, <0.01), platelet count (p=0.037, <0.05), the predisposition course of antibiotics before hospitalization (p=0.037, <0.05), and gender (p=0.044, <0.05) are respectively correlated with the hospitalization course. For Spearman correlation analysis between the hospitalization course versus α diversity, β diversity, and the abundances of certain species at the phylum or genus level, α diversity (Shannon) (r=-0.520, p=0.001, <0.01), β diversity (Bray–Curtis dissimilarity) (r=-0.413, p=0.011, <0.05), the abundances of *Firmicutes* (r=0.336, p=0.042, <0.05), *Proteobacteria* (r=-0.357, p=0.030, <0.05) at the phylum level, abundances of *unclassified_Enterobacteriaceae* (r=-0.448, p=0.005, <0.01), and *Escherichia_Shigella* (r=-0.386, p=0.018, <0.05) at the genus level showed a significant association with the hospitalization course (**Figure 13**).

## Discussion

This study firstly compared the lung microbiome and the demographic characteristics of children with hematological malignancies and LRTIs versus children with LRTIs and no any malignancies. Between the two groups with age, weight, height, gender, and predisposed antibiotics course before hospitalization all matched, significant differences were found in the red blood cell count, hemoglobin, platelet count, C-reactive protein, ratio of positive culture other than bronchoalveolar lavage fluid(BALF) and hospitalization course. Among microbiome of all BALF samples from the two groups, LRTI children with hematological malignancies showed obviously decreased α and β diversity, significantly increased function in infectious disease:bacteria/parasite, drug resistance:antimicrobial and human pathogenesis than control group (LRTI children without any hematological malignancies), distinctly reduced proportion of *Firmicutes*, *Bacteroidota*, *Actinobacteriota*, increased *Proteobacteria* at the phylum level, and obviously elevated proportion of *Parabacteroides*, *Klebsiella*, *Grimontia*, *Escherichia_Shigella*, *unclassified_Enterobacteriaceae* at the genus level than the control group. Besides, it was revealed that α diversity(Shannon), β diversity(Bray Curtis dissimilarity), *Proteobacteria* at the phylum level, *unclassified_Enterobacteriaceae* and *Escherichia_Shigella* at the genus level, significantly negatively associated with hospitalization course, whereas *Firmicutes* at the phylum level established a positive correlation with hospitalization course.

Consistent with previous studies (23, 24), LRTI children with hematological malignancies were easier to get immunity and hemocyte exacerbations when attacked by LRTIs than other children with similar infectious conditions. In this study, children with malignancies turned out with significantly lower level of red blood cell count, hemoglobin, platelet count, and obviously higher level of C-reactive protein, ratio of a positive non-BALF culture, lower level of tidal volume per kilogram, and longer entire lower respiratory tract infection course and hospitalization course. Nonetheless, in a large cohort study of acute respiratory infections in children and adolescents with acute lymphoblastic leukemia, patients with viral LRTIs had a significantly lower nadir absolute lymphocyte count compared with those with viral upper respiratory tract infections (8). Further, larger sample–sized and multicentered studies are in great demand about this field. Moreover, children with malignancies showed a higher ratio of pulmonary infiltration other than bronchopulmonary infiltration, higher percentage of bilateral loci in CT, and pulmonary complications than in the control group. Given the results of this study, on one hand, the immunity system of children with hematological malignancies seemed more fragile on the account of chemotherapy and malignancies’ pathogenic mechanism. On the other hand, in previous studies and daily clinical work, common pathogens observed among children with hematological malignancies used to be variable from those among other children, with much stronger virulence and invasion ability than common community-acquired pathogens. It should be noted that in this study children with malignancies and LRTIs older than 6 years old seemed have equal pulmonary function with the age-matched control group, while children with malignancies and LRTIs under 6 years old showed significantly lower level of tidal volume per kilogram, a restrictive pulmonary dysfunction indicator, than the age-matched control group. Based on the previous studies about the worsening trend in pulmonary function for children with hematological malignancies (25–27), we have reason to believe that younger children with malignancies and LRTIs seem to worsen more in restrictive pulmonary dysfunction than age-matched children with only LRITs. This may be related with the more unstable and immature situation in younger children, yet more large cohort studies for this aspect are still greatly needed in the future.

α (within-subject) Diversity was found significantly decreased in LRTI children with hematologic malignancies than the control group, with both the abundance and OTUs lower than the control group. More frequent hospital visits, upper or lower tract infections, empirical antimicrobial treatment than other children, and even chemotherapy may account for this distinct difference. Unlike other studies about the common airway microbiome composition of other diseases (15, 28–31), in this study, LRTI children with hematological malignancies revealed higher abundance in *Proteobacteria*, yet lower abundance in *Firmicutes*, *Actinobacteriota*, and *Bacteroidota* at the phylum level and, at the genus level, obviously higher abundance in *Escherichia_Shigella*, *unclassified_Enterobacteriaceae, Klebsiella*, and *Parabacteroides*, the majority of which were pathogens of intestinal flora, but lower abundance in *Streptococus, Rothia, and Bacteroides*. As reported in other studies (32, 33), there were always variable correlations between the abundances of certain species at different levels. In our study, *Proteobacteria* was obviously negatively associated with *Firmicutes* and *Bacteroidota*; in addition to that, *Firmicutes* and *Bacteroidota* were significantly positively correlated with each other. At the genus level, *Escherichia_Shigella* was significantly positively associated with *unclassified_Enterobacteriaceae, Klebsiella*, and *Parabacteroides*; in addition, *unclassified_Enterobacteriaceae* was obviously positively correlated with *Klebsiella.* Furthermore, *Klebsiella* versus *Parabacteroides* and *Streptococcus* versus *Rothia* both had a significantly positive correlation.

There were significant differences in the β (between-subject) diversity (Bray–Curtis) between two groups, the microbiome composition in BALF samples of LRTI children with hematological malignancies revealed high similarity within the group, while the composition in the samples of the control group showed more heterogeneity with each other within the group, which still have mutual similarity within the group and dissimilarity from the samples of the observation group (PERMANOVA, R:0.623, p:0.001, p<0.01). The biomarker analysis (LFeSe) of each group established that there were distinct species in each group eligible to be the reliable indicators. For the hematological malignancy group, *Proteobacteria* at the phylum level and *Parabacteroides*, *Klebsiella*, *Grimontia*, *Escherichia_Shigella*, and *unclassified_Enterobacteriaceae* at the genus level were validated. In addition, among the control group, *Firmicutes*, *Bacteroidota*, *Aenigmarchaeon*, and *Actinobacteriota* at the phylum level and *Caproiciproducens*, *Oceanobacillus*, *Bacillus*, *unclassified_Caloramatoracceae*, *Proteiniphilum*, and *Vibrio* at the genus level were good biomarkers. It turned out that microbial biomarkers for different diseases varied a lot (29, 31, 34–36), supporting that the tiny partially explored microbiome correlated with pathogenesis and progression of variable diseases work through numerous pathways.

As for function prediction analysis, BugBase phenotype prediction showed the lung microbiome from LRTI children with hematological malignancies having an elevated potentially pathogenic ability, facultatively anaerobic ability, and stress tolerance ability. For the KEGG metabolic pathway analysis, the function in drug resistance: antimicrobial, infectious disease:bacterial, and infectious disease:parasite was found obviously stronger in the microbiome from LRTI children with hematological malignancies. Furthermore, for FAPROTAX analysis, in terms of mammal_gut, human_gut, and human_pathogen_all, samples from LRTI children with hematological malignancies were indicated to be more superior than the control group. Altogether, the three kinds of microbiome function analysis in children with hematological malignancies were established with stronger microbial function in pathogenesis (bacteria or parasite) ability, survival ability even in unfavorable environments, and antimicrobial drug resistance ability, leading to more frequent, severe, and refractory respiratory infections in these children. In the UKPCCMP cohort (37), researchers did not identify any increased risk of severe/critical infection in children with hematological compared with non-hematological malignancies, consistent with the slightly decreased function in the infectious disease:viral of the microbiome from the observation group in our study. What cannot be ignored is that the microbiome from LRTI children with hematological malignancies showed stronger function in mammal_gut and human_gut to a certain extent, reminding us about the gut–lung axis theory of microbiomes (38–40).

About the correlation of clinical factors and microbiome α diversity and β diversity, the predisposition course of antibiotics before hospitalization was indicated to be associated with both α and β diversity. The easier choice for empirical antimicrobial drugs in front of febrile episodes in children with hematological malignancies may be the major cause to this situation, which was comprehensible and common nowadays, whereas, in this study, alanine transaminase revealed a significant correlation with α diversity, which was scarcely reported in previous studies (32, 41–43). There was a great need for further study about this term.

The hospitalization course was complicated with variable factors, such as disease severity, disease complications, disease pathogen, treatment plan, immunity condition, and adjuvant therapy, yet, in general, a hospitalization course could simply stand for the smoothness of disease management, consistent with other previous studies (28). For the correlation analysis of the hospitalization course and lung microbiome, it was established that the α diversity, β diversity, abundance of *Proteobacteria* at the phylum level, and abundance of *unclassified_Enterobacteriaceae* and *Escherichia_Shigella* at the genus level were negatively associated with the hospitalization course, whereas the abundance of *Firmicutes* was positively correlated with the hospitalization course. That indicated that the lower α diversity; β diversity; and the abundance of *Proteobacteria*, *unclassified_Enterobacteriaceae*, and *Escherichia_Shigella*, the longer and rougher the course and the higher payment would happen in LRTIs children.. However, the more *Firmicutes* children with LRTIs have in the lung microbiome, the more smoothly treating course they would have.

Taken together, what made the changes in this study existing were still unknown. There was evidence that smoking was shown to restrict the ability of alveolar macrophages to phagocytose and kill bacteria, changing the homeostasis of the lung microbiome (44). Children with hematological malignancies had chemotherapy for treatment and uncommon immunity conditions from the pathogenesis of hematological malignancies. Either of these two factors could do more harm than smoking to the lung microbiome, resulting in lung dysbiosis. Moreover, there is quantity evidence that the components of the enteric microflora, specifically Gram-negative bacilli, may also make up a component of the lung microflora (45, 46). The disruption of intestinal–pulmonary crosstalk is linked to increased susceptibility to airway diseases and infections (47). Furthermore, the concept of the gut–bone marrow–lung axis has gained increasing attention with the discovery of the influence of SCFAs on bone marrow hematopoiesis (48, 49). The latest study also exhibited that the lung microbiome regulates brain autoimmunity, breaking through the so-called tight security of the brain (50), which indicated that the lung microbiome could also affect other organs or systems. Therefore, changes in the gut microbiome or bone marrow, immunity cells, and so on could be the potential origin of lung dysbiosis in children with malignancies and LRTIs, which could also cause a chain reaction to other organs, systems, or back onto the gut and bone marrow. Anyhow, along with many researchers, we have been committed to continuous studies for discovering the final trick.

Our study has several important strengths. Firstly, we reported the microbiome features from 16 BALF samples from children with hematological malignancies complicated with LRTIs, with age, weight, height, gender, and disease severity–matched children as the control group(children with LRTIs without any basement diseases). We found distinctly different α diversity, β diversity, abundance of certain species in BALF samples from LRTI children with hematological malignancies, indicating that the lung microbiome of children with hematological malignancies developed a stronger ability in pathogenesis, antimicrobial drug resistance, and unfavorable environment tolerance. To some extent, we could take advantage of macrobiotic biomarker (α diversity, β diversity, the abundance of *Proteobacteria*, *unclassified_Enterobacteriaceae*, *Escherichia_Shigella*, or *Firmicutes*) tests to predict the hospitalization course according to the significant correlation between them.

There were limitations in this study. Firstly, the sample size was limited since the samples are not so easy to obtain. A future larger cohort with more samples from multiple airway loci in children will be of great value. Secondly, the data in this study were collected with one single center so that results may not be generalizable. Validation across centers is important. Thirdly, this study was limited to the lack of the gut microbiome data for deeper research. Further study including the lung and gut microbiome is under way to uncover whether gut dysbiosis is consistent with the lung’s and which system would be the origin of these changes. Fourthly, children with hematological malignancies complicated with LRTIs compared with the control group had hematological malignancies and chemotherapy as additional factors; the difference between two groups could not be simply ascribed to any single factor. We have begun a longitudinal observation on the sputum and oral swab of children at the time of initially being diagnosed as hematological malignancies and other certain time nodes during chemotherapy to support the conclusion in this study and promote further study in this field.

In summary, this study firstly characterizes the lung microbiome of children with or without hematological malignancies complicated with LRTI. LRTI children with hematological malignancies had a decreased α and β diversity; significantly reduced abundance of *Firmicutes*, *Bacteroidota*, *Actinobacteriota*; increased *Proteobacteria* at the phylum level; and distinctly elevated abundance of *Parabacteroides*, *Klebsiella*, *Grimontia*, *Escherichia_Shigella*, and *unclassified_Enterobacteriaceae* at the genus level, significantly increased function in infectious disease pathogenesis, antimicrobial drug resistance, and unfavorable environment tolerance, than the LRTI children without any malignancies. Furthermore, α diversity (Shannon), β diversity (Bray–Curtis dissimilarity), *Proteobacteria* at the phylum level, and *unclassified_Enterobacteriaceae* and *Escherichia_Shigella* at the genus level were significantly negatively associated with the hospitalization course whereas *Firmicutes* at the phylum level was positively correlated with the hospitalization course.

## Data availability statement

The original contributions presented in the study are publicly available. This data can be found here: https://doi.org/10.5061/dryad.wm37pvmr3.

## Ethics statement

This study was reviewed and approved by Qilu Hospital of Shandong University Institutional Review Board. Written informed consent to participate in this study was provided by the participants’ legal guardian/next of kin.

## Author contributions

YZ initiated the study, participated in the design and coordination, and drafted the manuscript. WYZ and HNN did equal roles in data management. JL provided statistical support. FHL and JFC helped to initiate the study and edit the manuscript. We are grateful to the entire research team. Finally, we are especially grateful to the patients and families whose data had been enrolled in the study. All authors contributed to the article and approved the submitted version.

## Funding

This research received grant from Natural Science Foundation for Youths of Shandong Province (ZR2020QH055) and Qilu Hospital of Shandong University Scientific Research Fund Youth Project (2017QLQN32).

## Conflict of interest

The authors declare that the research was conducted in the absence of any commercial or financial relationships that could be construed as a potential conflict of interest.

## Publisher’s note

All claims expressed in this article are solely those of the authors and do not necessarily represent those of their affiliated organizations, or those of the publisher, the editors and the reviewers. Any product that may be evaluated in this article, or claim that may be made by its manufacturer, is not guaranteed or endorsed by the publisher.
