# Supplementary figures and images for: Lung microbiome in children with hematological malignancies and lower respiratory tract infections
Source: Front Oncol. 2022 Sep 21;12:932709. doi: 10.3389/fonc.2022.932709 (PMC9533145; doi:10.3389/fonc.2022.932709)

**A**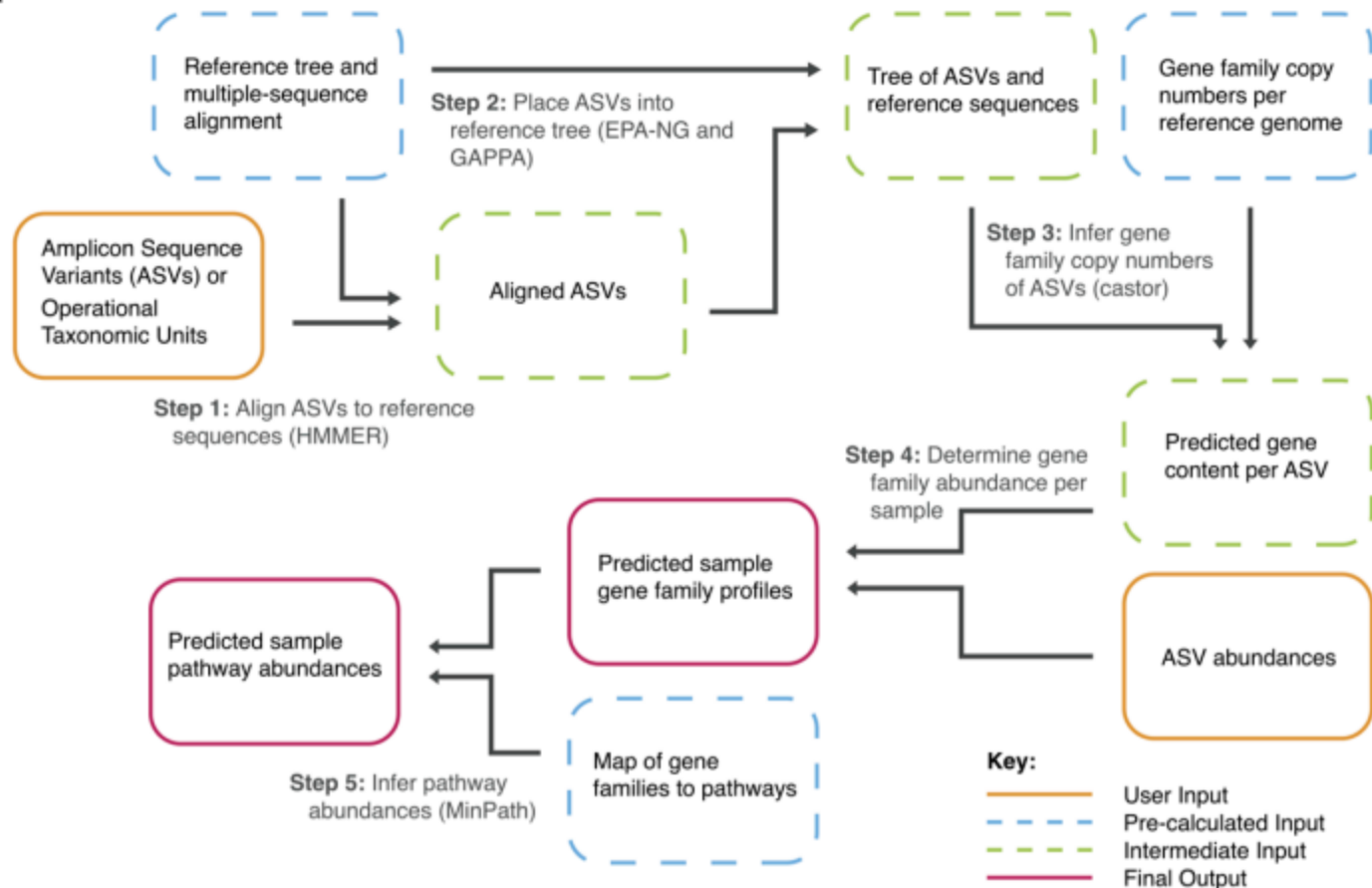

Supplement: Supplementary file 1 [file DataSheet_1.pdf]
