# Supplementary material for: Lung microbiome in children with hematological malignancies and lower respiratory tract infections
Source: Front Oncol. 2022 Sep 21;12:932709. doi: 10.3389/fonc.2022.932709 (PMC9533145; doi:10.3389/fonc.2022.932709)
Supplement: Supplementary file 2 [file Table_1.docx]

**Supplementary Table 1. Correlation between clinical factors and lung microbiome in LRTIs children with or without hematological malignancies**

| **Clinical Factors** | **P-value of correaltion with α diversity(Shannon)^*^** | **P-value of correaltion with β diversity(Bray Curtis dissimilarity)^*^** |
| --- | --- | --- |
| **Weight** | 0.901 | 0.672 |
| **Height** | 0.795 | 0.842 |
| **Age** | 0.503 | 0.370 |
| **Predisposition course of antibiotics before hospitalization** | **0.025** | **0.042** |
| **Hospitalization course** | 0.922 | 0.418 |
| **WBC** | 0.923 | 0.665 |
| **ANC** | 0.208 | 0.395 |
| **RBC** | 0.969 | 0.712 |
| **Hb** | 0.149 | 0.132 |
| **PLT** | 0.842 | 0.926 |
| **CRP** | 0.622 | 0.896 |
| **ALT** | **0.012** | 0.079 |
| **LDH** | 0.525 | 0.404 |

Abbreviations: WBC=white blood cells count; ANC=absolute neutrophil count; RBC=red blood cells count; Hb=hemoglobin; PLT=platelet; CRP=C reactive protein; ALT=alanine transaminase; LDH=lactate dehydrogenase.

*Statistically significant p values(p<0.05) are shown in bold.
